# Purification and characterization of a surfactant-compatible lipase from *Aspergillus tamarii* JGIF06 exhibiting energy-efficient removal of oil stains from polycotton fabric

**DOI:** 10.1007/s13205-016-0449-z

**Published:** 2016-06-10

**Authors:** Arijit Das, Srividya Shivakumar, Sourav Bhattacharya, Sujina Shakya, S. S. Swathi

**Affiliations:** Department of Microbiology, Center for Post Graduate Studies, Jain University, 18/3, 9th Main, Jayanagar, 3rd Block, Bangalore, 560011 Karnataka India

**Keywords:** *Aspergillus tamarii*, Lipase, Vegetable oil, Surfactant

## Abstract

An extracellular lipase with 23,666.66 U/ml/min activity was produced by *Aspergillus tamarii* JGIF06 under submerged fermentation in mineral salt medium containing coconut oil (2.5 % v/v), tryptone (2 % w/v) and ammonium chloride (2 % w/v), with initial pH of 5 ± 0.2, incubated at 25 °C for 7 days on a rotary shaker at 120 rpm. A 7.9-fold increase in lipase-specific activity was recorded after purification by DEAE Sepharose ion exchange and Sephadex G200 column chromatography. The apparent molecular mass of this enzyme was revealed as 50 kDa by sodium dodecyl sulphate polyacrylamide gel electrophoresis. The optimal lipase activity was recorded at pH 4 and 37 °C. The enzyme revealed broad specificity towards different vegetable oils. The *K*_*m*_ and *V*_max_ of the lipase on olive oil was found to be 330.4 mg and 53,690 U/ml/min, respectively. The lipase activity was stable in the presence of surfactants such as cetrimonium bromide, sodium dodecyl sulphate and Tween 80, and metal ions and reagents such as Ca^2+^, Ba^2+^ and 2-mercaptoethanol. However, the activity was greatly reduced in the presence of organic solvents such as chloroform. The stain removal potential of the crude lipase was determined on polycotton fabric pieces stained with peanut oil. Lipase added to cold water alone significantly enhanced the removal of stain by 152 %. The addition of lipase also improved the stain removal efficiency of a commercially available detergent in the presence of either cold (25 ± 2 °C) or hot (65 ± 2 °C) water. The current findings suggest the potentiality of this enzyme for energy-efficient biocatalytic application.

## Introduction

Lipases (triacylglycerol acylhydrolases, EC 3.1.1.3) are enzymes that catalyse the hydrolysis of triacylglycerol to glycerol and free fatty acids (Sharma and Kanwar 2014). Lipases from microorganisms are considered better than those obtained from plants or animals because of faster production, higher yields and greater stability. The enzyme production can be enhanced by selection of potent strains, genetic manipulation and optimization of growth conditions (Dayanandan et al. 2013).

High production cost of lipase is a major hurdle in its application in industrial processes. Therefore, various attempts have been made to lower the cost of its production (Smaniotto et al. 2012). Fungi are usually preferred as lipase producers because they synthesize extracellular enzymes that can be easily extracted from the production media (Maia et al. 1999). However, the synthesis of fungal lipases is governed by both nutritional and physico-chemical parameters such as pH, temperature and level of dissolved oxygen.

Some of the applications of lipases include synthesis of organic chemicals, flavour enhancement during cheese production and treatment of high fat-containing waste water (Sharma et al. 2001). Fungal lipases are extensively used in the detergent industries for removal of tough oily stains from fabrics. Rapid degradation of oils with better specificity even under mild conditions has made these enzymes useful in the formulation of soaps and production of biosurfactants (Gopinath et al. 2003). However, very few studies which discuss the characterization of surfactant-compatible fungal lipases are available. A detailed analysis of lipase properties facilitates its application in relevant industrial processes (Borrelli and Trono 2015). Owing to the continuous demand for fungal lipases in various industrial sectors, the present study was undertaken with the objectives of purification, characterization and determination of the oil-destaining potential of lipase from a selected fungal isolate.

## Materials and methods

### Source of fungal strain, substrates and chemicals

A lipolytic fungal strain, isolated from rhizospheric soil, was obtained from the Department of Microbiology, Center for Post Graduate Studies, Jain University, Bangalore, India. The lipolytic activity was monitored by observing a deep blue colour around the fungal colony on Spirit blue agar. The fungal isolate was identified as *Aspergillus tamarii* JGIF06 (GenBank Accession No. KR259959) by partial 18S rDNA sequencing at Chromous Biotech Pvt. Ltd., Bangalore, India. Pure culture of the fungus was maintained on potato dextrose agar slants and stored at 4 °C until use. All the analytical-grade chemicals and reagents were procured from HiMedia Laboratories Pvt. Ltd., Mumbai, India. The edible-grade vegetable oils and detergent powder were purchased from provisional stores in Bangalore, India.

### Production of lipase

Lipase production was carried out in 1000 ml Erlenmeyer flask containing 500 ml of supplemented mineral salts medium containing (g/l) tryptone, 20; NH_4_Cl, 20; K_2_HPO_4_, 3; KH_2_PO_4_, 1; MgSO_4_, 0.1; MgCl_2_, 0.12; cetrimonium bromide (CTAB), 0.5 % (w/v); coconut oil, 2.5 % (v/v) and distilled water, adjusted to an initial pH of 5 ± 0.2 and autoclaved. The sterile medium was aseptically inoculated with 1.5 % (v/v) fungal conidial suspension prepared in mineral salt solution and incubated at 25 °C for 7 days on a rotary shaker at 120 rpm. Post-incubation, the fungal broth was filtered using Whatman’s No. 1 filter paper and centrifuged at 5000 rpm for 30 min at 4 °C. The clear supernatant was subjected to lipase assay and further purification.

### Lipase assay

The enzymatic assay of lipase was performed by the titrimetric method using olive oil as the substrate and thymolphthalein as the indicator (Padhiar et al. 2011). The end point was determined by the change in the colour of the reaction mixture from colourless to pale blue. One unit of enzyme activity (U) was defined as µmol of free fatty acids released due to enzyme action and expressed as U/ml/min.

### Protein estimation

The protein content was evaluated using the method of Lowry et al. (1951) with 200 µg/ml of crystalline bovine serum albumin fraction V as the standard.

### Purification of lipase

The chilled clear supernatant containing crude lipase was subjected to ammonium sulphate precipitation till 80 % saturation was attained at 4 °C. The resulting precipitate was centrifuged at 5000 rpm for 30 min at 4 °C and resuspended in a minimum volume of 0.1 M phosphate buffer (pH 6) at 4 °C. The enzyme solution was dialysed using dialysis membrane 50 (Himedia, Mumbai, India) against 0.01 M phosphate buffer (pH 6) at 4 °C overnight. The dialysed enzyme was loaded onto DEAE Sepharose column (2 × 10 cm) pre-equilibrated with 20 mM Tris–HCl buffer (pH 6) and eluted with a linear gradient of (0–200 mM) NaCl in the same buffer at a flow rate of 0.5 ml/min. The eluted fractions showing lipase activity were pooled and applied to Sephadex G200 column (2 × 20 cm) pre-equilibrated with 20 mM Tris–HCl buffer (pH 6) at a flow rate of 0.5 ml/min. Fractions showing lipase activity were pooled and stored at 4 °C.

### Determination of molecular mass

The relative molecular mass of the purified lipase was determined by SDS-PAGE in a Mini Protean Tetra cell vertical electrophoresis unit (Bio-Rad) using a 10 % (w/v) acrylamide gel, following the method described by Laemmli (1970). Lipase samples (crude and purified) were analysed after staining with Coomassie Brilliant Blue R-250 and molecular mass was estimated with reference to medium range molecular weight protein marker (Genei, Bangalore, India).

### Zymography

After non-denaturing PAGE with the purified lipase, the gel was placed onto a pre-solidified plate containing 3 % (v/v) olive oil and 0.001 % (w/v) Rhodamine B in 2 % (w/v) agarose gel. After overnight incubation at 27 ± 2 °C, in situ lipase activity was detected as a fluorescent band under 350 nm UV light (Castro-Ochoa et al. 2005).

### Characterization of lipase

The optimum pH for lipase activity was measured using olive oil as the substrate, at 27 °C in buffers (200 mM) of different pH values such as citrate buffer (pH 3.0–5.0), phosphate buffer (pH 6.0–8.0) and glycine–NaOH buffer (pH 9.0–10.0). The optimum temperature for the lipase activity was determined at different temperatures (4, 27, 37, 45, 60, 80 and 100 °C) using 200 mM citrate buffer of pH 4. Lipase substrate specificity was analysed using different vegetable oils (coconut oil, peanut oil, sesame oil, sunflower oil, soybean oil and olive oil). The effect of substrate concentration on lipase activity was evaluated by incubating 1 ml of the enzyme with varying concentrations (460–9200 mg) of substrate (olive oil) at 37 °C. *K*_*m*_ and *V*_max_ values were calculated from Lineweaver–Burk plot using Hyper32 software.

The effect of surfactants on lipase activity was analysed after pre-incubating the enzyme for 15 min at 37 °C with 1 % concentration of detergents such as Triton X-100, Tween 80, Tween 20, CTAB or sodium dodecyl sulphate (SDS). The effect of diverse chemicals on lipase activity was determined after pre-incubation of the enzyme for 15 min at 37 °C with 10 mM concentration of CaCl_2_, MgCl_2_, MnCl_2_, ZnCl_2_, BaCl_2_, HgCl_2_, EDTA or 2-mercaptoethanol. The effect of organic solvents on lipase activity was determined after pre-incubation of the enzyme (0.2 ml) for 15 min at 37 °C in 0.5 ml of methanol, ethanol, 2-propanol, acetone or chloroform (Castro-Ochoa et al. 2005). Suitable controls were also maintained without the addition of any surfactant, metal salt or solvent. The residual activity was measured by titrimetric assay and expressed as percentage.

### Determination of oil-destaining efficiency

The crude fungal lipase was tested for its ability to remove oil stains from fabrics. Polycotton fabric was cut into pieces (3 cm × 2 cm) and each piece was stained with two drops of peanut oil used for deep frying. The fabric pieces were allowed to dry and subjected to various oil-destaining treatments such as plain water; water and commercially available detergent 1 % (w/v); water and lipase; water, lipase and detergent. Each of these treatments was studied using cold (25 ± 2 °C) or hot (65 ± 2 °C) water separately. The oil-stained fabric pieces were separately soaked in each treatment solution taken in a 100 ml Erlenmeyer flask and gently agitated. Post-incubation for 30 min, the fabric pieces were removed, dried and observed for residual oil stains. The efficiency of lipolytic activity was evaluated by the lipase titrimetric assay estimating the amount of free fatty acids released during each treatment.

### Statistical analysis

Lipase characterization was conducted in triplicate and the data have been graphically presented as mean ± standard deviation (SD) (*n* = 3). Oil-destaining studies were conducted in triplicate and the data were analysed by two-way Analysis of Variance (ANOVA) using GraphPad Prism version 6.

## Results and discussion

### Lipase production by *A. tamarii* JGIF06

Based on the results of media optimization (data not shown), lipase production by the lipolytic fungus *A. tamarii* JGIF06 was carried out using mineral salt medium supplemented with coconut oil, tryptone and NH_4_Cl. A high lipase activity (23,666.66 U/ml/min) was recorded in the culture filtrate. The positive impact of coconut oil on lipase production by *A. tamarii* JGIF06 might be attributed to its high content (92 %) of saturated fatty acids (primarily triglycerides). About 70 % of these are short- and medium-chain saturated fatty acids (Ribeiro et al. 2011). The short- and medium-chain fatty acids are utilized by fungi through the carnitine-independent transport system (Hynes et al. 2006; Son et al. 2012). Khoramnia et al. (2013) also reported maximum lipolytic activity from *Geotrichum candidum* when coconut oil was used as the substrate.

A combination of organic (tryptone) and inorganic (NH_4_Cl) nitrogen supplements in the production medium facilitated a high yield of lipase. This might be due to faster assimilation of inorganic nitrogen source by the fungal mycelia and enrichment of the production medium with several growth factors and amino acids derived from the organic nitrogen source. The current finding is in agreement with Bindiya and Ramana (2012) who reported that among various inorganic sources, NH_4_Cl supplementation showed the best lipase production from olive oil by *Aspergillus sydowii*.

Incorporation of cationic surfactant CTAB in the medium probably altered the cell permeability, thereby enhancing the enzyme secretion. A similar result was reported by Polizelli et al. (2008), who observed that the activity of lipase from *Pachira* *aquatica* was significantly enhanced in the presence of CTAB.

### Purification of lipase

The crude fungal lipase obtained by filtration of the fungal broth was clarified by centrifugation. The clear supernatant was concentrated by ammonium sulphate precipitation and desalted by dialysis. The partially purified enzyme showed a 3.4-fold increase in specific activity in comparison to the crude lipase, with a 50.7 % recovery. This was purified by DEAE Sepharose ion exchange chromatography and Sephadex G200 gel filtration chromatography. The purification steps resulted in a 7.9-fold increase in specific activity; however, the final recovery was 43.1 %. The purification profile of the fungal lipase is shown in Table 1.

**Table 1:** Purification profile of lipase from *A. tamarii* JGIF06

Purification step	Total protein (mg)	Total activity (U/ml/min)	Specific activity (U/mg)	Recovery (%)	Purification fold
Crude enzyme filtrate	0.71852	23,666.66	32,938.06	100	1
Ammonium sulphate precipitated fraction	0.26666	23,166.66	86,877.14	97.8	2.6
Dialysed fraction	0.10741	12,016.66	111,876.54	50.7	3.4
DEAE Sepharose and Sephadex G200	0.03920	10,200.25	260,210.46	43.1	7.9

### Molecular mass of lipase and zymography

**Fig. 1:**
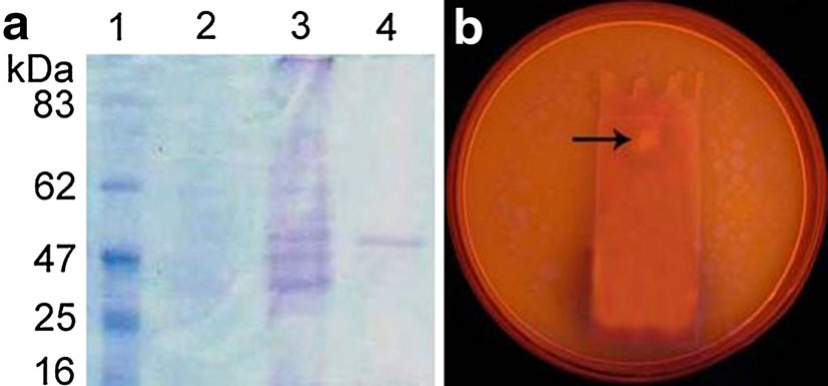
**a** SDS-PAGE profile of lipase from *A. tamarii* JGIF06. *1* Protein marker. *2* Uninoculated medium. *3* Crude lipase extract. *4* Purified lipase. The molecular sizes of the marker proteins are shown on the *left*. **b** Zymography of purified lipase showing a band of lipolysis (indicated by *arrow*) on visualization of gel under 350 nm UV light

The SDS-PAGE profile of crude lipase demonstrated multiple bands with relative molecular masses ranging between 25 and 62 kDa, whereas the purified lipase showed a single protein band, indicating that the apparent molecular mass is ~50 kDa (Fig. 1a). This is in close proximity with that reported by Padhiar et al. (2011), wherein an extracellular lipase from *Aspergillus flavus* showed an apparent molecular mass of 47 kDa. Ulker and Karaoğlu (2012) reported the production of an extracellular lipase from *Mucor hiemalis f. corticola* having a molecular mass of 46 kDa. Zymography was performed with the purified lipase using a non-denaturing gel. The gel was placed on agarose plate containing olive oil and Rhodamine B. When irradiated with UV light (350 nm), a clear orange band was observed which indicated lipolytic activity (Fig. 1b).

### Characterization of lipase

#### Effect of pH

**Fig. 2:**
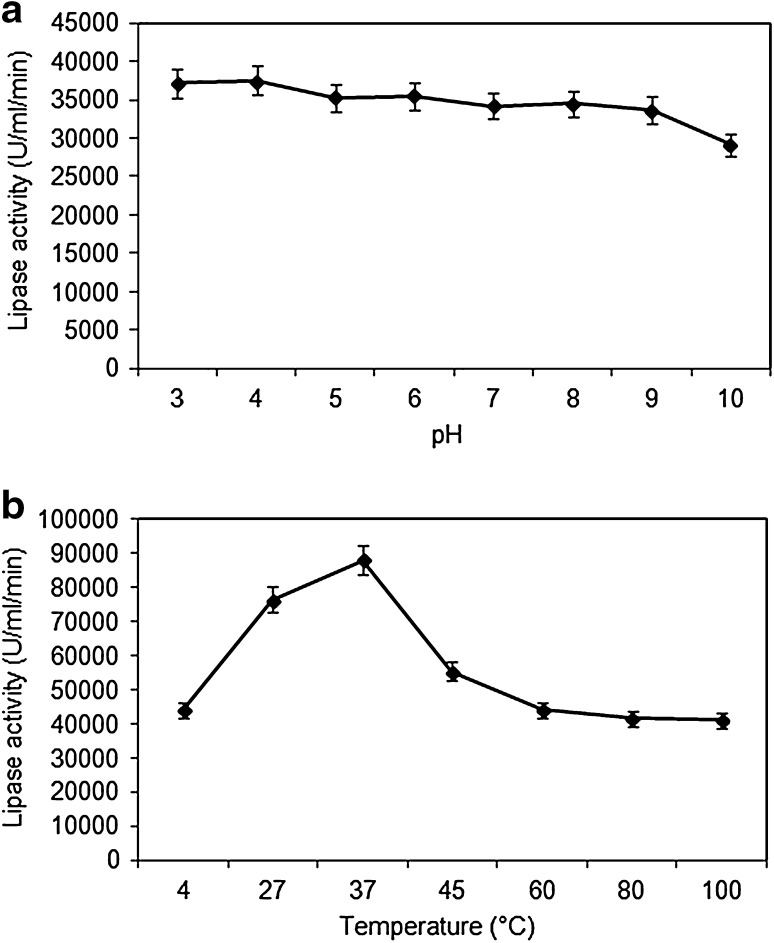
**a** Effect of pH on lipase activity. **b** Effect of temperature on lipase activity. Data represent mean ± SD (*n* = 3); *p* < 0.05

Enzymes are most active at their optimum pH, as their active sites have maximum interaction with the substrate. Any drastic change in the pH of a medium leads to denaturation of the enzyme resulting in the loss of its activity. Although the lipase from *A. tamarii* JGIF06 was active over a wide range of pH ranging from 3 to 9, maximum activity (37,500 U/ml/min) was recorded at pH 4 (Fig. 2a). At pH 10, the activity of the enzyme slightly decreased. Hiol et al. (1999) had reported that the lipase produced by *Mucor hiemalis f. hiemalis* was stable for 15 min in the pH range of 4–9.

#### Effect of temperature

Temperature plays a pivotal role in determining the activity of enzymes. 37 °C was found to be the optimum temperature for lipase activity (88,166.66 U/ml/min). Thereafter, an increase in temperature showed a gradual decline in the enzyme activity (Fig. 2b). The probable reason for this decrease might be due to the disruption of the tertiary structure of lipase which would have altered the configuration of the active site, thereby decreasing the enzyme substrate interaction. The enzyme retained 63 and 50 % of its activity at 45 and 60 °C, respectively. The current finding is supported by Hiol et al. (2000) who observed that the optimum temperature for activity of a lipase from *Rhizopus oryzae* was 35 °C, and 65 % of its activity was retained after 30 min of incubation at 45 °C.

### Substrate specificity of the lipase

**Fig. 3:**
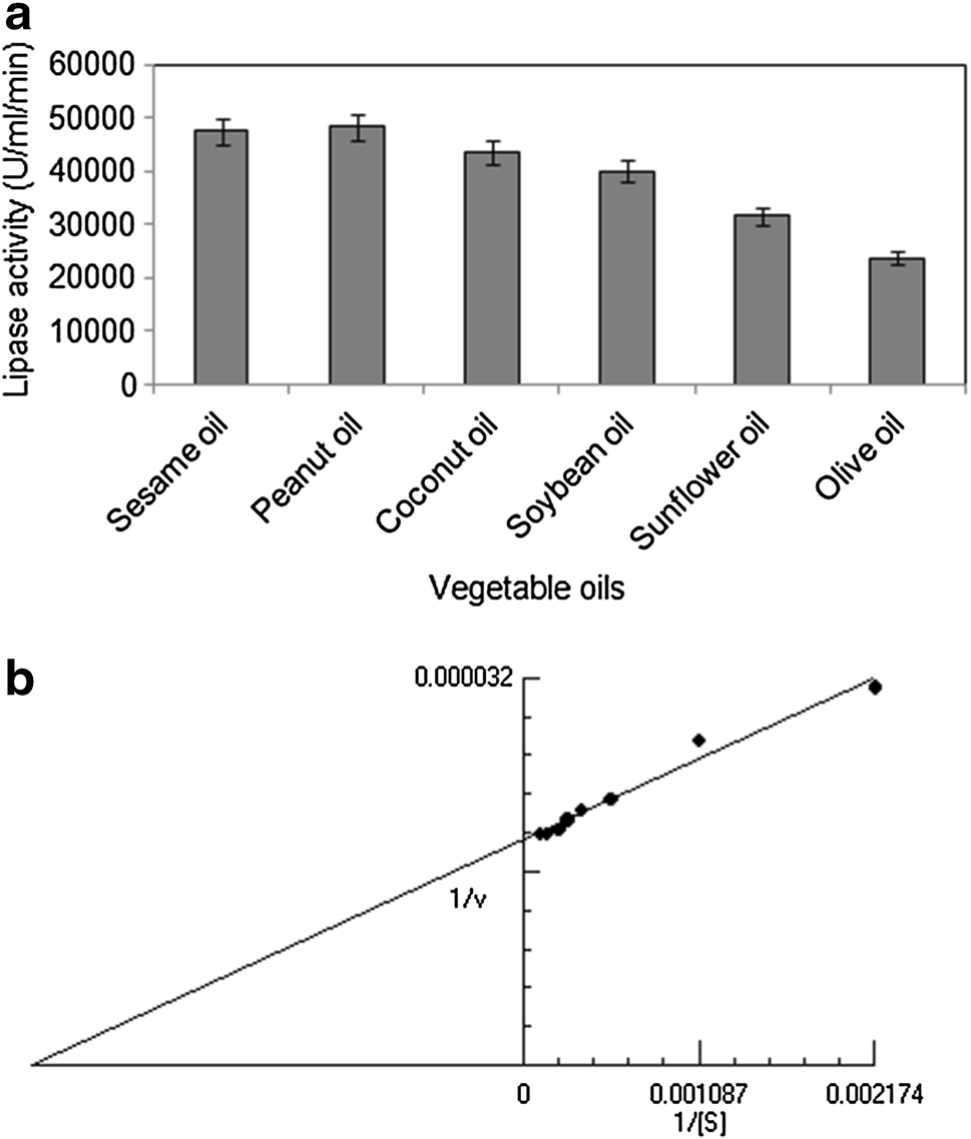
**a** Specificity of lipase towards different vegetable oils used as substrates. **b** Lineweaver–Burk plot using olive oil as substrate for the lipase. Data represent mean ± SD (*n* = 3); *p* < 0.05

Six different vegetable oils were analysed for determining the substrate specificity of the lipase. Among these, maximum lipase activity was revealed with peanut oil (48,333.33 U/ml/min) followed by sesame oil and coconut oil (Fig. 3a). The results of substrate specificity clearly indicated the versatile ability of this fungal lipase to act on different vegetable oils with moderately high contents of unsaturated fatty acids (75–80 % in sesame and peanut oils) as well as those with very high content of saturated fatty acids (86 % in coconut oil). A study conducted by Rahman et al. (2006) reported that the lipase produced by *Pseudomonas* sp. strain S5 had more affinity towards long-carbon chain natural oils like olive oil, groundnut oil, sesame oil and coconut oil. However, Cihangir and Sarikaya (2004) reported that an isolate of *Aspergillus* sp. showed better lipase activity when olive oil was used as the substrate.

### Effect of substrate concentration

An enzymatic reaction involves the interaction of the active site of an enzyme with its specific substrate. As the substrate concentration increases, the active sites of the enzyme molecules get saturated. Since the standard lipase assay was conducted using olive oil, the effect of substrate concentration on lipase activity was determined through Michaelis–Menten equation and Lineweaver–Burk plot using olive oil as the substrate (Fig. 3b). The *K*_*m*_ and *V*_max_ of the lipase from *A. tamarii* JGIF06 were found to be 330.4 mg and 53,690 U/ml/min, respectively. The low *K*_*m*_ value indicated that a small quantity of olive oil could saturate the enzyme and therefore the enzyme is specific for the substrate. The *K*_*m*_ and *V*_max_ of a lipase from psychrotrophic *Penicillium chrysogenum* 9′ were reported as 2.33 mM and 22.1 U/ml, respectively, using tributyrin as substrate (Bancerz et al. 2005).

#### Effect of surfactants

**Fig. 4:**
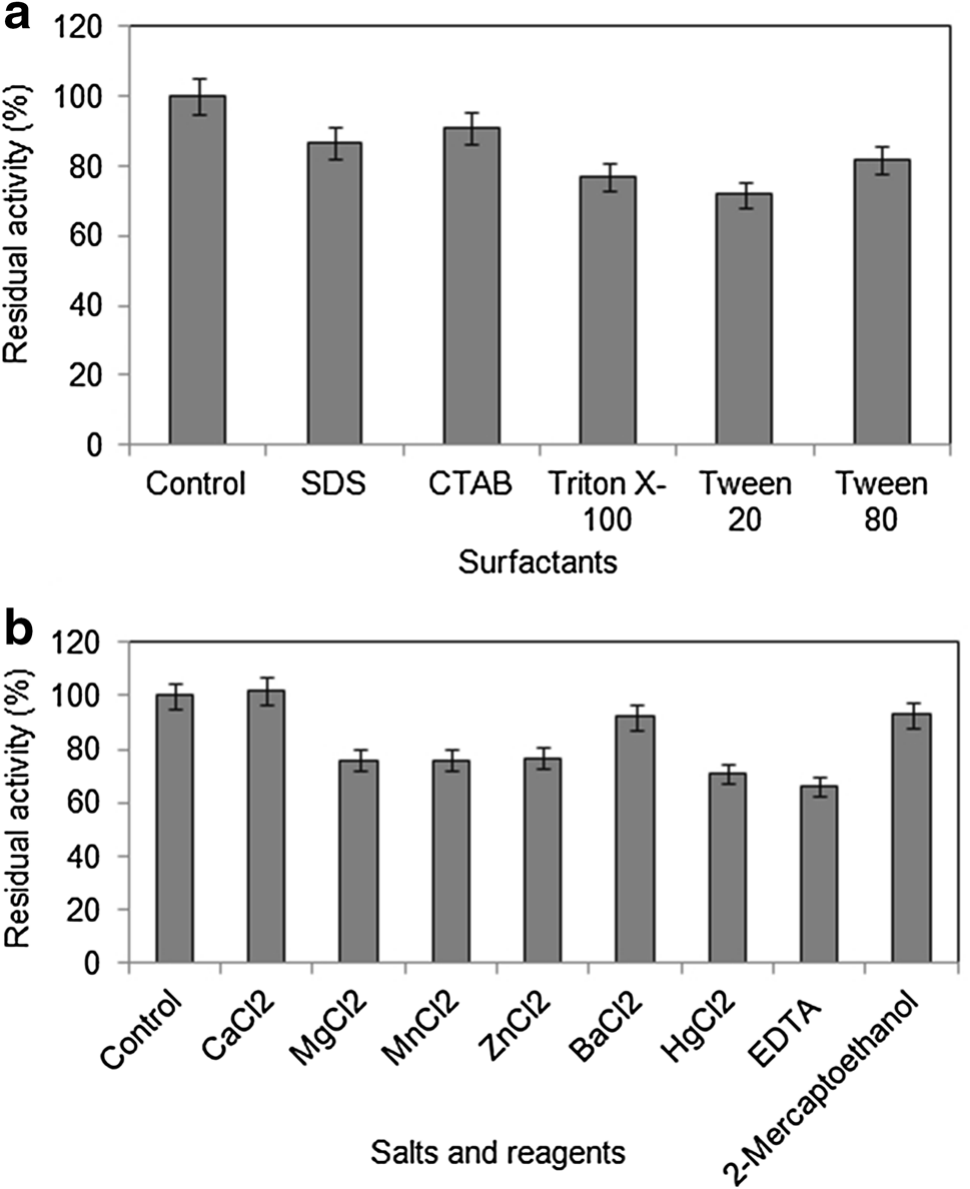
**a** Effect of surfactants on lipase activity. **b** Effect of metal salts and reagents on lipase activity. Data represent mean ± SD (*n* = 3); *p* < 0.05

Surfactants usually denature enzymes through disruption of their tertiary structures. The lipase from *A. tamarii* JGIF06 retained 90.84 % of its activity in the presence of CTAB, while the lowest residual activity was recorded with Tween 20 (71.83 %), compared to 100 % activity shown by the control (without any surfactant) (Fig. 4a). The results indicated the potentiality of the lipase to tolerate various surfactants like SDS, CTAB and Tween 80. The activity of a lipase produced by endophytic fungus *Cercospora kikuchii* was stable in the presence of surfactants such as Tween 20, Tween 80, SDS and Triton X-100, wherein 98–100 % of enzymatic activity was retained (Costa-Silva et al. 2014).

#### Effect of salts and reagents

Enzymes require metal ions as co-factors in various metabolic pathways. The results showed a slight increase in the enzyme activity in the presence of CaCl_2_, whereas 92 % activity was retained with BaCl_2_ (Fig. 4b). With the other metal ions, the residual lipase activities ranged between 71 and 76 %. Chartrain et al. (1993) observed that a lipase from *P. aeruginosa* MB 5001 was stimulated (1.24-fold) by adding 10 mM CaCl_2_, but was strongly inhibited by 1 mM ZnSO_4_ (94 % inhibition). In this study, the residual lipase activity was minimum (66.19 %) when pre-incubated in 10 mM EDTA. EDTA probably chelated the metal ions present with the enzyme, thereby denaturing the enzyme. Interestingly, the activity of the lipase was relatively stable in the presence of 2-mercaptoethanol. The resistance of the fungal lipase to 2-mercaptoethanol might indicate the presence of a lesser number of disulphide bonds exposed on the enzyme surface. A similar observation was made by Yadav et al. (1998) who reported that lipase from *A. terreus* was unaffected by 2-mercaptoethanol and potassium ferrocyanide.

#### Effect of organic solvents

Pre-incubation of the fungal lipase with each of the organic solvents showed a drastic reduction in its activity (in the range of 3.5–7.7 %) as compared to the control (100 %). The activity decreased in the following order: 2-propanol > acetone > ethanol > methanol > chloroform. The reduction in lipase activity when incubated with organic solvents might be due to the dehydrating action of organic solvents which would have removed water molecules from the vicinity of the enzyme, thus precipitating the enzyme which adversely affected its activity. In addition, all the organic solvents might have caused denaturation of the amino acid residues present in the enzyme molecule. Hernández-Rodríguez et al. (2009) reported that the activities of lipases from *Rhizopus* sp. were reduced in the presence of polar solvents like ethanol and *i*-propanol.

### Oil-destaining efficiency of the lipase

**Fig. 5:**
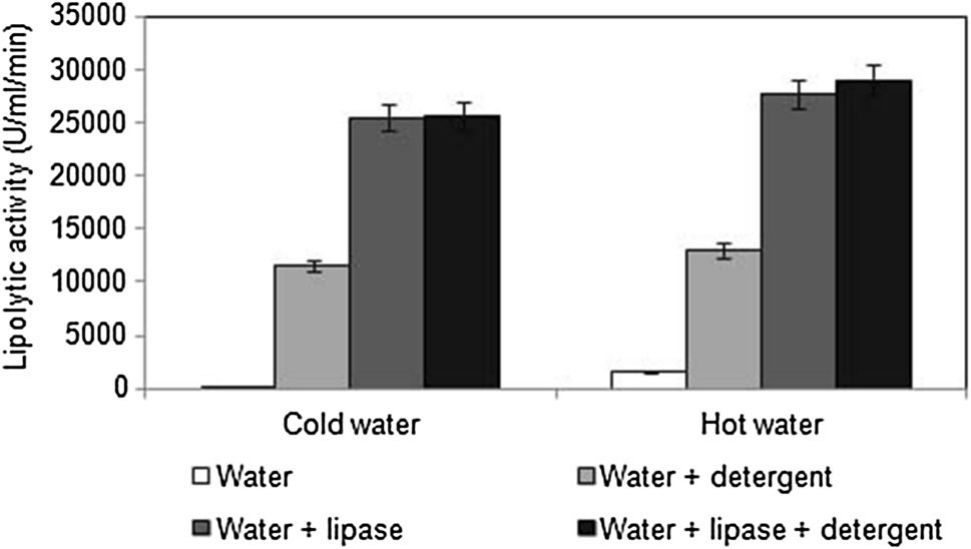
Oil-destaining efficiency of various lipolytic treatments on oil-stained fabric pieces. Data represent mean ± SD (*n* = 3); *p* < 0.05

Polycotton fabric pieces were stained with peanut oil. Peanut oil is usually preferred for deep frying because it is relatively stable with a high smoke point (Das et al. 2013). This explains the rationale behind the use of peanut oil in the present study to obtain tough stains on the fabric pieces. The oil-stained fabric pieces were subjected to different combinations of lipolytic treatments involving cold or hot water, detergent and lipase. Oil-destaining activity was least for the treatment involving only plain cold water, indicating its inefficiency (166.66 U/ml/min) (Fig. 5). Treatment with hot water alone did not promote efficient removal of oil stains. However, an increase in lipolytic activity was recorded when combinations of cold or hot water and detergent were used. Furthermore, even in the absence of detergent, the lipolytic activity was significantly enhanced by 152 and 17.4 % on addition of crude fungal lipase to cold and hot water, respectively. Treatments involving cold or hot water, lipase and detergent showed further enhancement in lipolytic activity, with the highest destaining potential (29,000 U/ml/min) recorded with hot water. Following various lipolytic treatments, it was evident that addition of the crude lipase resulted in significant enhancement in stain removal efficiency of a commercially available detergent compared to lower lipolytic activity (13,000 U/ml/min) and stain removal capacity of hot water with only detergent. This result clearly indicated the detergent-compatible nature of the fungal lipase. However, the lipolytic activities observed following various treatments with hot water did not reveal any significant difference from those obtained with cold water. It could be interpreted that the crude lipase was effective in removing oil stains even at moderately cold temperature, thereby reducing energy consumption. This indicates the possibility of its application in energy-efficient systems. Hemachander and Puvanakrishnan (2000) had reported that in the presence of a detergent, a lipase produced by *Ralstonia pickettii* enhanced the removal of oil by 24–27 % compared to treatment with only detergent. In an earlier study, a lipase from *Fusarium oxysporum* was found compatible with various surfactants and commercial detergents (Prazeres et al. 2006).

## Conclusions

The extracellular lipase produced by *A. tamarii* JGIF06 exhibited specificity towards a range of saturated and unsaturated vegetable oils. This may indicate its ability to remove a variety of lipid stains from fabric pieces. The stability in the presence of metal ions, surfactants and broad pH range, together with its lipolytic potential at moderate temperature, suggests the application of this lipase in detergent formulation and energy-efficient biocatalysis.
